# Sol–gel synthesis of Cu/Sn-doped TiO_2_ printed films with calcination-tuned interfaces for visible-light degradation of methylene blue and 4-chlorophenol

**DOI:** 10.1039/d6ra03562k

**Published:** 2026-08-10

**Authors:** Atasheh Soleimani-Gorgani, Jamal Al-Sabahi, Gayaneh Vartanyan, Mohammed Al-Abri, Zahra Al-Sharji, Behzad Shirkavand Hadavand

**Affiliations:** a Department of Printing Science and Technology, Institute for Color Science and Technology Tehran 16765654 Iran asoleimani@icrc.ac.ir; b Central Instrumentation Laboratory, College of Agricultural and Marine Sciences, Sultan Qaboos University 34, Al-Khoud 123 Oman; c Nanotechnology Research Center, Sultan Qaboos University Al-Khoud 123 Oman; d Department of Resin and Additives, Institute for Color Science and Technology P. O. Box: 16765-654 Tehran Iran

## Abstract

Efficient water treatment requires photocatalytic systems that combine high activity with scalable fabrication. In this work, Cu/Sn-doped TiO_2_ nanostructured interfaces were developed *via* a sol–gel method followed by fabrication of printed films to create tailored catalytic surfaces for pollutant removal from water. Since this photocatalyst is printed as a film, it remains fixed on the substrate and can be easily separated from the reaction medium. Co-doping with Sn^4+^ and Cu^2+^ modified the electronic structure and charge transfer behavior, as confirmed by photoluminescence, Raman spectroscopy, and EDX analysis. The photocatalytic performance was evaluated using methylene blue under UV irradiation and 4-chlorophenol under visible light. Among the prepared samples, the catalyst calcined at 500 °C exhibited the best performance, achieving approximately 90% degradation of methylene blue under UV light and about 41% degradation of 4-chlorophenol in aqueous solution under visible irradiation. UPLC analysis confirmed the progressive transformation of aromatic intermediates originating from 4-chlorophenol along the oxidative degradation pathway. This enhanced activity is attributed to improved crystallinity and effective dopant incorporation. These results demonstrate that combining dopant engineering with printed nanostructured films provides a promising and scalable approach for developing photocatalytic materials for water purification applications.

## Abstract 2

Efficient water treatment requires photocatalytic systems that combine high activity with scalable fabrication. Image created with the assistance of Biorender AI.

## Graphical abstract

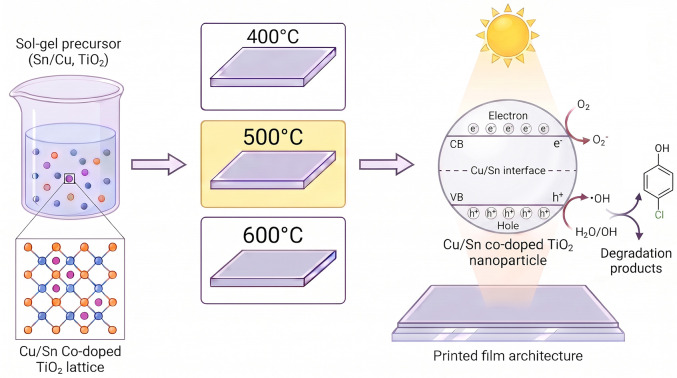

## 1 Introduction

Access to safe and clean water continues to be a critical challenge for societies, particularly in rapidly urbanizing and industrializing regions where aquatic ecosystems are increasingly exposed to complex organic contaminants and pharmaceutical residues.^1^ These pollutants often resist conventional treatment methods, necessitating the development of more advanced and sustainable water purification technologies. Among emerging strategies, solar-assisted photocatalysis has gained considerable interest due to its environmentally friendly nature, low operating costs, and potential for decentralized applications that operate independently of energy-intensive infrastructure.^2^ As such, it represents a promising tool for addressing water treatment needs in resource-limited settings while contributing to broader water sustainability objectives.^3^ Heterogeneous photocatalysis is especially valuable in this context, offering the ability to mineralize persistent organic pollutants under light-driven conditions.^4^

Titanium dioxide (TiO_2_) is one of the most thoroughly studied photocatalysts owing to its high chemical stability, low toxicity, and affordability.^5^ However, the intrinsic wide bandgap of anatase TiO_2_ (∼3.2 eV) considerably limits its activity towards ultraviolet (UV) light, which constitutes only a small portion of the solar spectrum.^6^ This limitation significantly reduces its effectiveness in solar-driven systems in wastewater treatment as a photocatalyst, which rely primarily on visible light. As a result, various modifications have been explored to enhance the optical absorption window and charge carrier dynamics of TiO_2_. One of the most effective strategies involves doping TiO_2_ with metal or non-metal ions to narrow the bandgap and introduce intermediate electronic states.^7^ Transition metal dopants such as Sn^4+^ and Cu^2+^ have shown particular promise in extending the spectral response of TiO_2_ and improving its photocatalytic activity. Sn^4+^, having an ionic radius close to that of Ti^4+^, can substitute directly into the lattice, reduce the bandgap, and suppress charge recombination through the creation of electron trap sites.^8^ Cu^2+^, on the other hand, contributes redox-active sites that facilitate charge transfer and enhance the generation of reactive oxygen species (ROS), thereby promoting photocatalytic reactions.^9^

When introduced simultaneously, these dopants are expected to act synergistically, improving light absorption and charge carrier separation more effectively than single doping. The practical realization of such co-doped photocatalysts also depends on the synthesis approach. The sol–gel method offers notable advantages for producing doped TiO_2_ nanoparticles, including low-temperature processing, cost-effectiveness, and the ability to control dopant dispersion and nanoparticle size.^10^ Its solution-based chemistry is also compatible with scalable fabrication methods such as printed-layer architectures, which ensure uniform catalyst distribution and maximize surface exposure to light. These printed films hold growing promise for integration into engineered water treatment systems designed for real-world operation.

Despite significant progress in doping strategies and photocatalyst synthesis, relatively few studies have combined both co-doping and printed-layer fabrication in a unified platform geared toward practical application. In particular, the engineering potential of Cu/Sn-doped TiO_2_ integrated into printed-layer systems remains underexplored, especially in terms of scalability, light utilization efficiency, and real-world applicability under solar light conditions.^11^ There is a clear need to bridge the gap between material development and process engineering by creating photocatalytic systems that are not only effective in lab-scale settings but also compatible with low-cost, large-area, and potentially field-deployable treatment platforms. Such an approach would directly support efforts toward sustainable and modular water purification technologies.

In this study, Cu/Sn-doped TiO_2_ nanoparticles were synthesised *via* a sol–gel process and thermally treated at three different temperatures to optimize their structural and surface properties. The resulting materials were then incorporated into printed-layer photocatalytic films to ensure uniform dispersion and enhanced light exposure. Since this photocatalyst is printed and not in powder form, it does not disperse into the medium, and thus there is no concern about recovering it from the medium. The photocatalytic performance of these systems was investigated using representative organic pollutants under both UV and visible light. By integrating material innovation with process-focused design, this work aims to contribute to the advancement of scalable and energy-efficient photocatalytic water treatment systems tailored to modern environmental engineering challenges.

## 2 Experimental

### 2.1 Materials

**Fig. 1:**
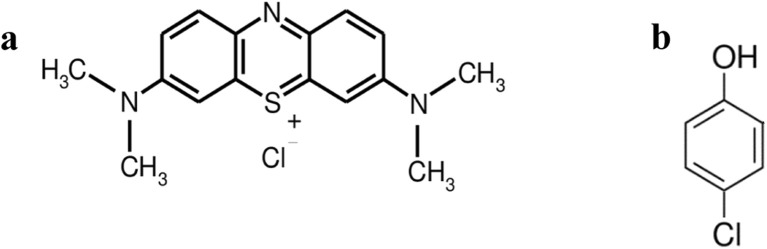
Chemical structures of: (a) methylene blue and (b) 4-chlorophenol.

All chemicals were used as received, with no additional purification or filtration. Deionized water was used in all experimental steps. Titanium(iv) isopropoxide (TTIP, 97%) and isopropyl alcohol (≥99.5%) were purchased from Sigma-Aldrich, while copper(ii) acetate (≥98.0%) and tin(iv) chloride (≥98%) came from Merck, Germany. Methylene blue (MB) and 4-chlorophenol (≥98%) served as model pollutants in this study; the former was obtained from a local textile factory (analytical grade), and the latter from Merck. The chemical structures are presented in Fig. 1.

### 2.2 Synthesis of Cu/Sn-doped TiO_2_ nanoparticles

Cu/Sn-doped TiO_2_ nanoparticles were synthesised using a sol–gel method according to a previously reported procedure.^9^ Titanium(iv) isopropoxide (8 mL) was dissolved in isopropyl alcohol (60 mL) and stirred for 15 min to obtain a clear precursor solution. Copper(ii) acetate (0.01 g) and tin(iv) chloride (0.01 g), previously dissolved in deionized water, were then added to the titanium precursor solution under continuous stirring. Subsequently, controlled hydrolysis was initiated by the dropwise addition of deionized water, leading to the formation of a homogeneous sol. The resulting sol was stirred at room temperature for 2 h to promote condensation and gel formation. The washed gel was dried at 100 °C for 12 h and subsequently calcined at 400, 500, and 600 °C for 2 h with a heating rate of 10 °C min^−1^.

### 2.3 Paste preparation and screen printing

A printable paste was prepared by dispersing 1.2 g of optimally synthesised Cu/Sn-doped TiO_2_ nanoparticles in a mixture of 2-propanol, α-terpineol, ethyl cellulose, and appropriate additives.

Glass substrates were cleaned by sequential ultrasonication in deionised water, acetone, methanol, and 2-propanol prior to use. The paste was deposited using an automated screen printer (SP1400, Sepas Co., Iran) equipped with a 100-mesh screen and a square-edged polyurethane squeegee. The films were printed in three successive passes, yielding a thickness in the range of 0.6–0.7 µm, and subsequently heat-treated at 500 °C for 1 h.

### 2.4 Characterization of the synthesised nanomaterial

The crystalline structure of the nanoparticles was analyzed using X-ray diffraction (XRD) with a Philips PW1730 instrument, employing Cu Kα radiation (*λ* = 0.15406 nm). Diffraction patterns were recorded over a 2*θ* range of 10° to 80° to assess the mineral composition. Photoluminescence (PL) spectra of the synthesised nanoparticles were measured using a UV-VIS-NIR fluorescence spectrophotometer (PerkinElmer Lambda-25) under ultraviolet excitation at 348 nm. Raman spectroscopy was conducted using an XploRA Plus system with a 785 nm laser, capturing spectra in the range of 100–3500 cm^−1^. The surface morphology of the Cu/Sn-doped TiO_2_ nanoparticles was examined by field-emission scanning electron microscopy (FE-SEM, MIRA3, TESCAN, Czech Republic).

### 2.5 Evaluation of photocatalytic activity

The test sample was exposed to UV light using a 6 W lamp with a peak emission at 253.7 nm—a wavelength chosen due to the strong absorbance of most UV-responsive materials around 254 nm. To avoid interference from ambient light, the entire setup was enclosed in aluminum foil. The reaction took place in a glass column measuring 0.5 cm in diameter and 30 cm in length. To assess the photocatalytic performance of the nanocatalysts in degrading methylene blue (MB) under UV light, a 10 ppm MB solution was prepared, and 100 mL of this solution was used for each test. The Cu/Sn-doped TiO_2_ nanoparticles as a printed film was added to the solution. The photocatalytic activity was examined both under UV irradiation and in the absence of light (dark conditions) for comparison. The extent of methylene blue degradation was monitored by measuring absorbance at 664 nm with a UV-vis-NIR spectrophotometer. The degradation efficiency was calculated based on the reduction in methylene blue concentration, as described by eqn (1).^12^

1 % Degradation = ((Pollutant_in_ − Pollutant_out_)/Pollutant_in_) × 100

The photocatalytic degradation of 4-chlorophenol was conducted at room temperature under simulated sunlight irradiation. The photocatalytic degradation experiments were performed under simulated sunlight irradiation using a solar simulator (Sciencetech SS1.6 kW, London, ON, Canada) with a controlled irradiance of 1000 W m^−2^. The light source was positioned 30 cm from the reaction vessel and provided an emission wavelength range of 200–2500 nm. To evaluate the performance of the synthesised Cu/Sn-doped TiO_2_ nanoparticles as a printed film, ultra-performance liquid chromatography (UPLC, LC-30AD, Shimadzu, Japan) was used to monitor the breakdown of 4-chlorophenol and identify any intermediate by-products formed during the reaction. The degradation efficiency was calculated by using eqn (1).

## 3 Results and discussion

### 3.1 X-ray diffraction (XRD)

**Fig. 2:**
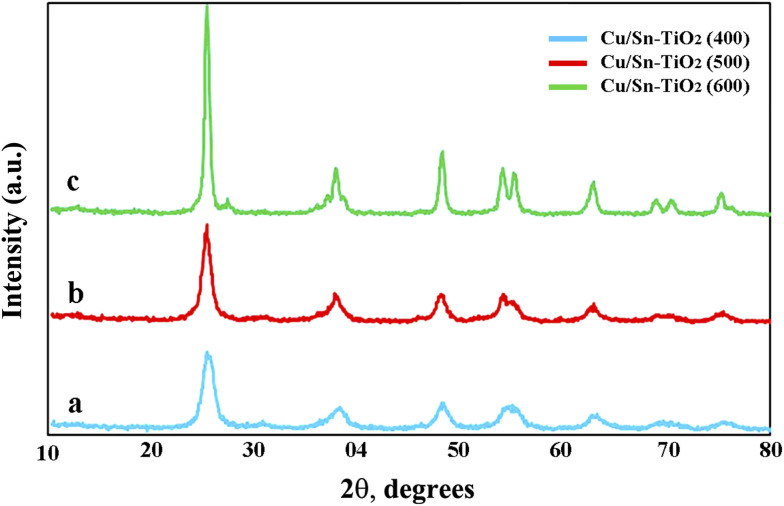
XRD patterns of nanoparticles calcined at different temperatures (a) 400 °C; (b) 500 °C and (c) 600 °C.

The XRD pattern of the Cu/Sn-doped TiO_2_ catalyst is presented in Fig. 2. The diffraction peaks observed at 25.4° (101), 38.73° (004), 48.25° (200), 54.1° (105), and 55.2° (211) correspond to the anatase phase of TiO_2_, in accordance with JCPDS card no. 21-1272. Peaks at 43.6° and 75.06° indicate the presence of metallic copper, while the characteristic peaks at 62.70° and 68.78° suggest the existence of amorphous CuO. Additionally, small peaks at 36.9° and 38.6° reveal traces of metallic tin (Sn).^11^ A diffraction peak at 70.06° was attributed to a hexagonal quartzite-like phase, likely corresponding to SnO. The crystallite size of the samples was estimated using the Scherrer equation, as given in eqn (2).^9^

2 *Φ* = *Kλ*/*β* cos *θ*

where *Φ* is the crystallite size; *K* is the shape factor with a value close to unity; *λ* is the wavelength of X-rays; *β* is the full width half-maximum (FWHM) of the main intensity peak; and *θ* is the Bragg angle.

Eqn (2) was used to calculate the average crystallite size (Table 1). For any photocatalytic application the need for improved visible light activity and high temperature stability are two fundamental desirable properties. In this study, the XRD results show the presence of mixed phase at a high calcination temperature of 500 °C by doping very minute fraction of Cu and Sn. The size of the crystals also decreases with increasing calcination temperature of the nano catalyst. This reduction in crystallite size is proposed due to segregation of the dopant cations at the grain boundary which inhibits the grain growth by restricting direct contact of grains.^13^ The formation of crystallites of rutile phase at a low calcination temperature is induced by the presence of Sn and Cu as previously reported. Metals may be finely dispersed, incorporated in the TiO_2_ crystal structure, or crystals are too small for detection due to a relatively low metal doping. Absence of a peak corresponding to Cu/Sn metals may be due to low metal ion dopants content and appropriate dispersion of metal ions on TiO_2_ crystal structure.^13^

**Table 1:** The average crystal size of nanoparticles calcined at different temperatures

Sample	Crystal size (nm)	Phase structure	Peak pos.	*E*_g_ (eV)
Cu/Sn-doped-TiO_2_ (400 °C)	55.4	A: 100, R: —	25.30	2.2
Cu/Sn-doped-TiO_2_ (500 °C)	33.1	A: 94, R: 6	25.27	2.1
Cu/Sn-doped-TiO_2_ (600 °C)	18.4	A: 91, R: 9	25.32	2.14

The phase contents of the samples are calculated by the following eqn (3):^13^

3 Rutile phase% = 100/[1 + 0.08(*I*_A_/*I*_R_)]

where *I*_A_ and *I*_R_ are integrated intensities of the anatase and rutile peaks, respectively.

### 3.2 Photoluminescence spectroscopy (PL)

**Fig. 3:**
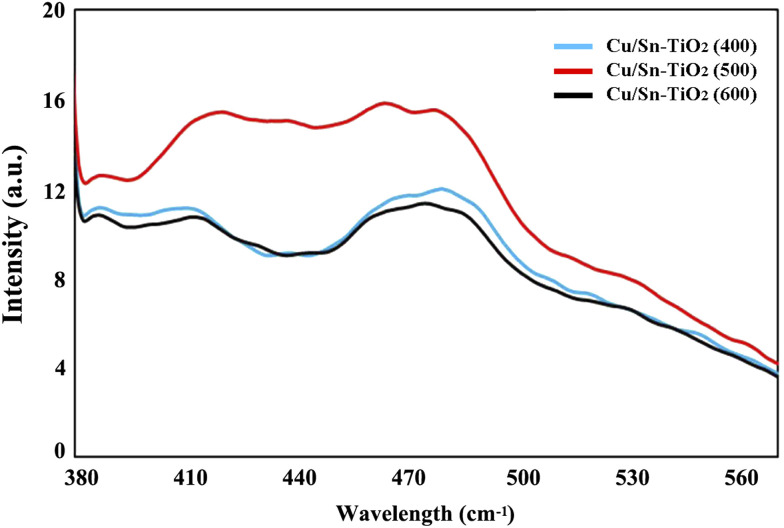
Photoluminescence (PL) spectrum of the synthesised nanoparticles calcined at different temperatures and pristine TiO_2_.

Fig. 3 shows the photoluminescence (PL) spectra of pristine TiO_2_ and Cu/Sn-doped TiO_2_ and nanoparticles, providing insight into thair electronic structure and optical behavior. Samples were excited at 348 nm; pristine TiO_2_ exhibits an emission peak at 411.5 nm. All samples show strong absorption below 500 nm, attributable to interband electronic transitions. In the Cu/Sn–TiO_2_ sample calcined at 500 °C, the characteristic TiO_2_ emission at ∼411.5 nm is substantially attenuated and appears only as a shoulder, while the emission centered near 470 nm shifts to lower energy. These spectral changes indicate a reconfigured defect landscape and an altered band structure following co-doping. The higher PL intensity observed for the 500 °C sample relative to pristine TiO_2_ is consistent with defect engineering induced by Cu and Sn incorporation, which is known to generate oxygen vacancies and localized defect states within the bandgap. Previous studies report that Sn incorporation into TiO_2_ enhances PL intensity by creating impurity levels and oxygen vacancies that modify radiative recombination pathways; such defect states can act as intermediate energy levels and produce enhanced broadband emission even when charge separation improves.^14–19^ Conversely, Cu doping has been shown in some reports to reduce PL intensity.^20,21^ Therefore, the net PL response in our Cu/Sn co-doped samples likely reflects the competing effects of Sn- and Cu-related defect mechanisms.

The bandgap energy (*E*_g_) can be estimated by eqn (4):

4 *αhν* = *A*(*hν* − *E*_g_)^*n*^

where *α* is the absorbance coefficient, *h* is the Planck constant, *ν* is the wave number, *A* is a constant and *E*_g_ is the bandgap energy in which *n* = 1/2 for direct bandgap materials and *n* = 2 for in indirect bandgap.^22^

XRD analysis revealed a decrease in crystallite size with increasing calcination temperature, which may result from segregation of Cu and Sn at grain boundaries that restricts grain growth during thermal treatment. This trend is accompanied by an increased rutile fraction at higher calcination temperatures; because rutile TiO_2_ has a narrower bandgap than anatase, the observed phase transformation can contribute to a reduced *E*_g_ and altered optical properties.^12,23^

### 3.3 Raman spectroscopy

**Fig. 4:**
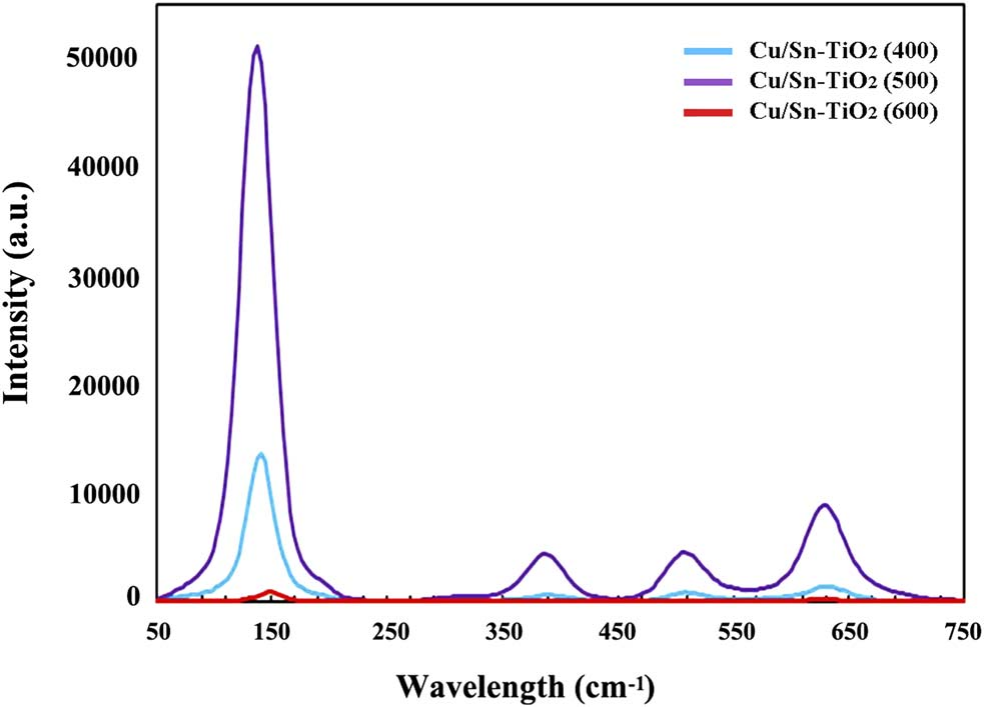
Raman spectra of nanoparticles calcined at different temperatures.

The Raman spectra of Cu/Sn-doped TiO_2_ nanoparticles are shown in Fig. 4. The observed vibrational bands at 147 cm^−1^ (E_g_), 199 cm^−1^ (E_g_), 399 cm^−1^ (B_1g_), 518 cm^−1^ (A_1g_ + B_1g_), and 643 cm^−1^ (E_g_) confirm the presence of the anatase phase in undoped TiO_2_ calcined at 400 °C.^17^ The inset in Fig. 4 highlights the precise peak positions (in cm^−1^) for samples, showing that the most intense peak in the doped samples shifts from 147 cm^−1^ to around 140 cm^−1^. Notably, the characteristic Raman bands of crystalline SnO_2_ –typically found at 490, 574, 636, and 776 cm^−1^– were absent, indicating that tin is not present as a separate crystalline oxide phase. This observation aligns well with the XRD results. The Raman spectra of Cu/Sn-doped TiO_2_ also show a red shift in all doped samples, suggesting no formation of rutile or other secondary phases. The consistent shift from 147 cm^−1^ to 140 cm^−1^ in doped samples is likely due to the successful incorporation of Sn^4+^ and Cu^2+^ ions into the TiO_2_ lattice.

### 3.4 Field emission scanning electron microscopy (FESEM)

**Fig. 5:**
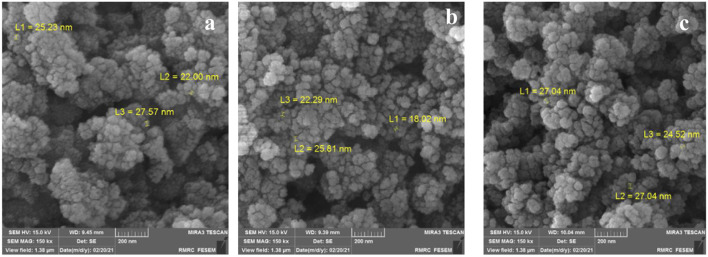
FESEM images of nanoparticles calcined at different temperatures (a) 400 °C; (b) 500 °C; (c) 600 °C.

The structure and surface morphology of the synthesised Cu/Sn-doped TiO_2_ nanoparticles were further examined using scanning electron microscopy (FESEM). Fig. 5 presents FESEM micrographs of the catalyst synthesised at different calcination temperatures. The images suggest a trend of decreasing average particle size with increasing temperature; however, this observation is not conclusive.

**Fig. 6:**
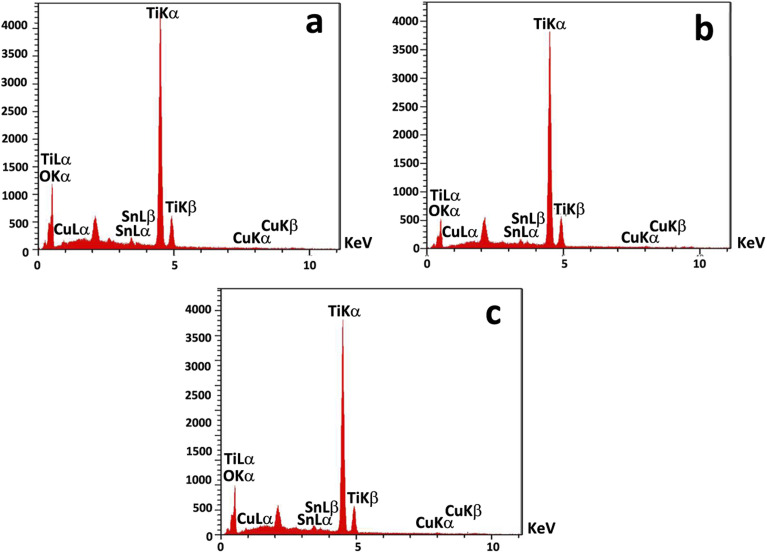
EDX Analysis of nanoparticles calcined at different temperatures (a) 400 °C; (b) 500 °C and (c) 600 °C.

EDX analysis confirmed the presence of copper (Cu) and tin (Sn) on the surface of TiO_2_. The spectra shown in Fig. 6 clearly display the characteristic peaks of Cu, Sn, and Ti. Peaks at 4.5 keV and 4.9 keV correspond to titanium, while the presence of copper is indicated by peaks at 8.04 keV and 0.93 keV. Tin is identified by peaks at 3.63 keV and 3.85 keV. The distribution of Cu and Sn was found to be uniform across the TiO_2_ surface. The EDX spectra of the Cu/Sn/TiO_2_ catalysts are presented in Fig. 6, and the elemental composition results are summarized in Table 2.

**Table 2:** Elemental analysis for Cu/Sn-doped TiO_2_ nanoparticles

Element	Cu/Sn-doped TiO_2_ (400 °C)	Cu/Sn-doped TiO_2_ (500 °C)	Cu/Sn-doped TiO_2_ (600 °C)
Ti	46.57	30.16	70.51
O	50.92	67.13	28.76
Cu	0.62	1.01	0.31
Sn	1.89	1.70	0.42

### 3.5 Photocatalytic degradation of methylene blue and 4-chlorophenol by Cu/Sn-doped TiO_2_

**Fig. 7:**
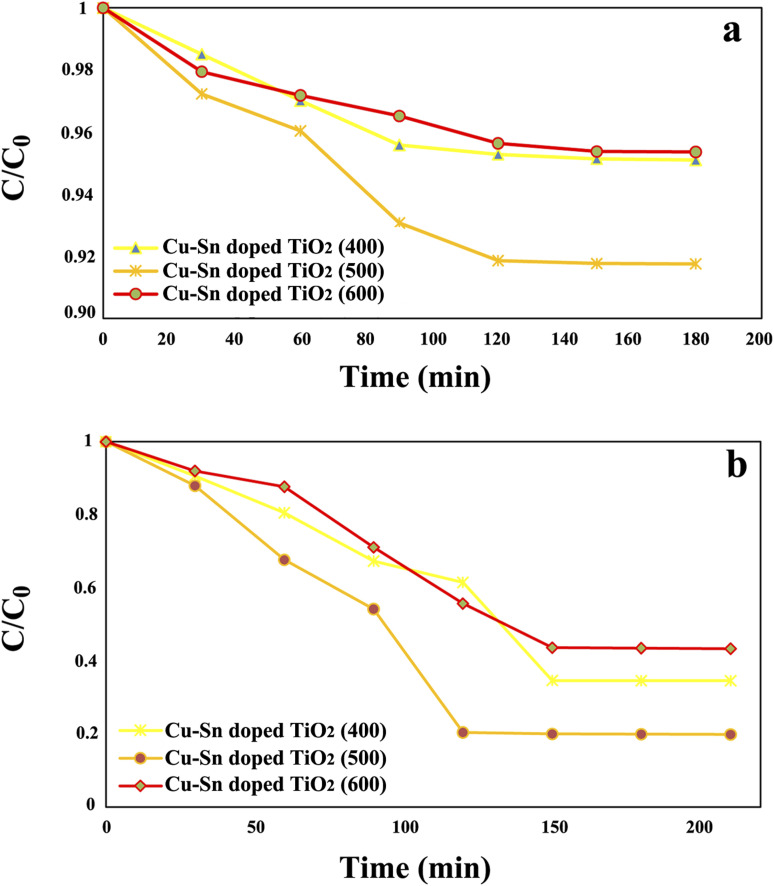
Photocatalytic degradation curves of methylene blue: (a) without light irradiation, (b) under UV light irradiation.

The photocatalytic performance of the printed Cu/Sn-doped TiO_2_ nanocatalysts was evaluated through the degradation of methylene blue (MB). Fig. 7 compares the photocatalytic activity of samples calcined at different temperatures under UV irradiation and in dark conditions. Under both conditions, the activity follows the order: 500 °C > 400 °C > 600 °C. The superior performance of the sample calcined at 500 °C under UV light can be attributed to an optimal balance between crystallinity, defect density, and dopant incorporation.

At 400 °C, the incomplete phase transformation of TiO_2_ limits the effective incorporation of Cu and Sn into the lattice, hindering the formation of shallow electron traps that facilitate charge separation, and thus reducing photocatalytic efficiency. In contrast, calcination at 500 °C promotes improved crystallinity and more effective dopant integration, while maintaining a relatively high surface area that enhances pollutant adsorption and interfacial charge transfer.^24^ Additionally, recent studies have shown that increased surface area enhances pollutant adsorption by improving contact between TiO_2_ and contaminants. At higher temperatures (600 °C), grain growth and possible dopant segregation reduce the active surface area and may introduce recombination centers, leading to diminished activity.

**Fig. 8:**
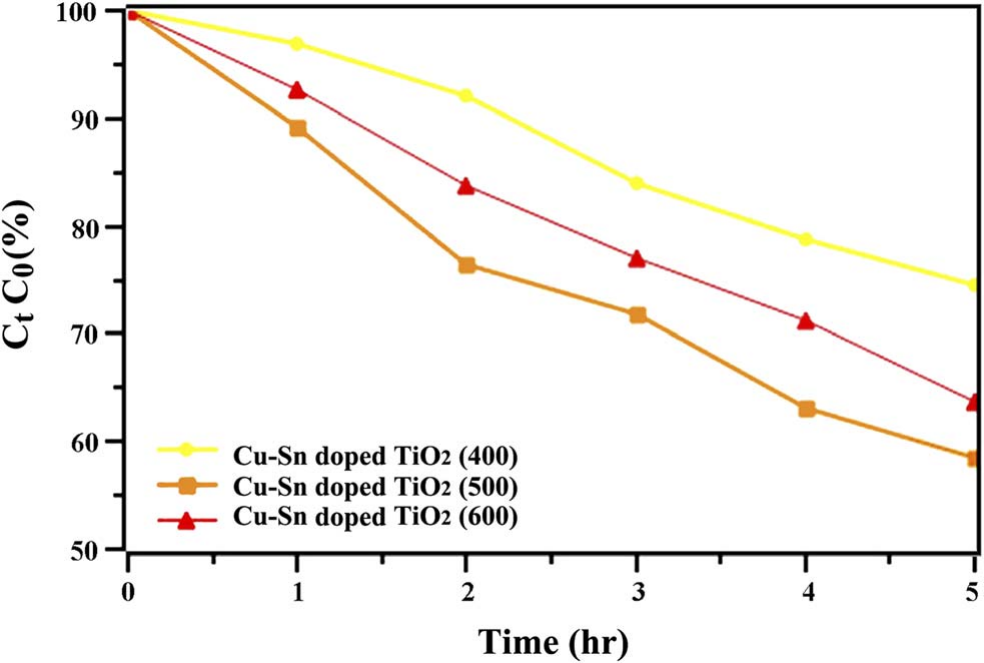
Photocatalytic degradation of 4-chlorophenol using printed layer of Cu/Sn-doped TiO_2_ irradiated with visible light.

The photocatalytic activity of the printed nanocatalysts was further examined for the degradation of 4-chlorophenol under visible light Fig. 8. The sample calcined at 500 °C again exhibited the highest performance, achieving approximately 41% degradation, compared to 25% and 36% for the samples treated at 400 °C and 600 °C, respectively. The sample calcined at 500 °C exhibited the highest PL intensity among all samples. It should be noted that PL intensity does not necessarily show an inverse correlation with photocatalytic activity.^14–19^ Accordingly, despite its high emission intensity, the 500 °C sample demonstrated superior photocatalytic performance, highlighting the critical roles of structural optimization, interfacial charge-transfer dynamics, anatase/rutile heterojunctions, and dopant-induced defects, rather than PL intensity alone.

To clarify the degradation pathway of 4-chlorophenol, the formation of intermediate products was monitored during irradiation. The degradation intermediates (acetic acid, benzoquinone, hydroquinone, and catechol) were identified under the same conditions by comparison with analytical standards. Each standard was analyzed under identical UPLC conditions and its UV spectrum was recorded over the 200–400 nm range to confirm the identity of the detected intermediates. Under visible light, hydroxyl radicals (˙OH) initiate attack on the aromatic ring, leading to two main reaction routes: one through hydroquinone, which can convert to benzoquinone in the presence of hydroxide ions, and another through catechol intermediates. Both routes ultimately led to the formation of acetic acid as a relatively benign intermediate, which was further oxidized, suggesting progression toward mineralization.^25^

**Fig. 9:**
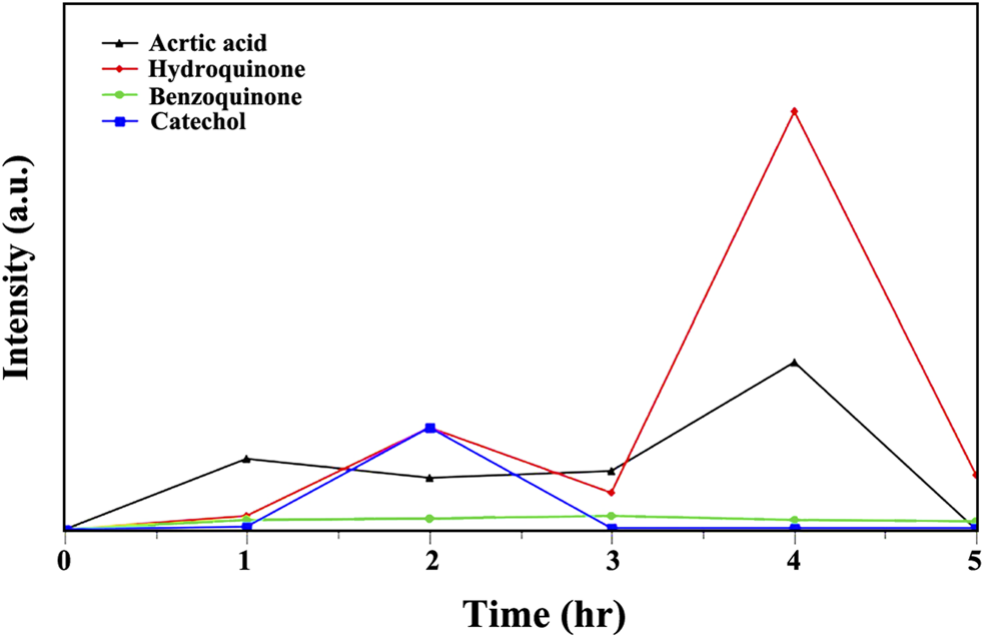
Intermediate by-products detected at various stages of photocatalytic degradation of 4-chlorophenol under visible light irradiation with one-layer printed Cu/Sn-doped TiO_2_ (calcined at 500 °C) used as a photocatalyst.

As shown in Fig. 9, hydroquinone initially accumulates and then stabilizes after about four hours, followed by a sharp decrease. Benzoquinone remains at low levels throughout the reaction. Catechol reaches a maximum at around two hours and declines markedly by three hours, indicating that both pathways operate simultaneously. Acetic acid peaks at approximately four hours and nearly disappears by five hours, confirming the catalyst's effectiveness in driving the reaction to completion.

### 3.6 Reusability of the printed photocatalyst for 4-chlorophenol degradation

The reusability of the printed Cu/Sn-doped TiO_2_ nanocatalyst was evaluated for the degradation of 4-chlorophenol under visible light irradiation. The photocatalytic performance was investigated over three consecutive cycles (5 h per cycle) to assess the stability and practical applicability of the system. The Cu/Sn-doped TiO_2_ film calcined at 500 °C was used for repeated photocatalytic tests. After each cycle, the film was carefully recovered, rinsed with deionized water, dried, and reused under identical conditions.

**Fig. 10:**
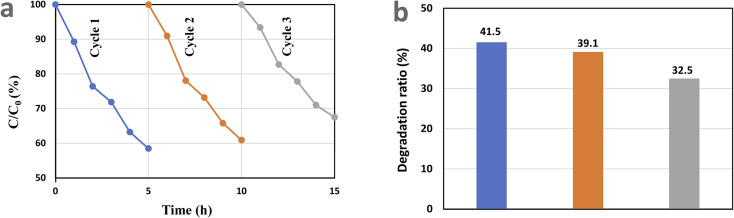
Reusability performance of the printed Cu/Sn-doped TiO_2_ (500 °C) nanocatalyst; (a) the efficiency of the photocatalytic degradation of 4-chlorophenol after three cycles; (b) degradation ratio after 5 cycles.

As depicted in Fig. 10, the photocatalytic efficiency decreased by only approximately 9% after the third cycle, demonstrating the good stability and reusability of the printed catalyst.

## 4 Conclusion

This study demonstrates that strategic surface engineering can convert a conventional photocatalyst into a functional, printable interface for solar-driven water remediation. By co-doping TiO_2_ with Sn and Cu and integrating the resulting nanoparticles into additively manufactured films, we engineered a nanostructured surface that balances visible-light absorption, charge separation, and structural stability. Among the synthesized nanoparticles, the sample calcined at 500 °C showed the most favorable performance, not only in terms of degradation efficiency but also in reflecting the critical role of calcination temperature in governing crystallinity, dopant distribution, and surface morphology. Together, these parameters define the interfacial environment in which light absorption, charge transfer, and pollutant oxidation occur.

Importantly, the printed architecture provides uniform catalyst exposure and mechanical cohesion, and may enable reuse in practical applications. Beyond the performance data, the results indicate that co-doping modifies interfacial electronic states, suppresses photoluminescence, and controls phase evolution. Overall, this study supports a surface-centered photocatalytic strategy in which engineered printed interfaces, rather than conventional powder catalysts, offer a scalable and potentially deployable route for water treatment. Reusability was systematically evaluated, showing that the photocatalyst retains its activity over multiple cycles with only about a 9% decrease in performance.

## Author contributions

Atasheh Soleimani-Gorgani: conceptualization, project administration, methodology, investigation, writing – review and editing. Jamal Al-Sabahi: investigation, methodology, writing – review and editing. Mohammed Al-Abri: investigation, methodology. Gayaneh Vartanyan: investigation, methodology, writing – original draft. Zahra Al-Sharji: investigation, methodology. Behzad Shirkavand Hadavand: investigation, writing – review and editing.

## Conflicts of interest

The authors declare that they have no known competing financial interests or personal relationships that could have appeared to influence the work reported in this paper.

## Data Availability

All data and materials supporting the results reported in this article have been deposited in the Zenodo repository and are openly available at https://doi.org/10.5281/zenodo.21597241. The repository includes the raw and processed data corresponding to the figures and tables presented in the article, as well as relevant experimental details required for reproducibility, in accordance with the journal's data-sharing policy.
